# Inspiratory muscle strength training improves weaning outcome in failure to wean patients: a randomized trial

**DOI:** 10.1186/cc10081

**Published:** 2011-03-07

**Authors:** A Daniel Martin, Barbara K Smith, Paul D Davenport, Eloise Harman, Ricardo J Gonzalez-Rothi, Maher Baz, A Joseph Layon, Michael J Banner, Lawrence J Caruso, Harsha Deoghare, Tseng-Tien Huang, Andrea Gabrielli

**Affiliations:** 1 Department of Physical Therapy, University of Florida, 1600 South West Archer Road, PO Box 100154, Gainesville, FL, 32610, USA; 2 Department of Physiological Sciences, University of Florida, 1600 South West Archer Road, PO Box 100144, Gainesville, FL, 32610, USA; 3 Department of Medicine, Division of Pulmonary, Critical Care and Sleep Medicine University of Florida, 1600 South West Archer Road, PO Box 100225, Gainesville, FL, 32610, USA; 4 Department of Anesthesiology, Division of Critical Care Medicine, University of Florida, 1600 South West Archer Road, PO Box 100254, Gainesville, FL, 32610, USA; 5 Department of Surgery, University of Florida, 1600 South West Archer Road, PO Box 100129, Gainesville, FL, 32610, USA

## Abstract

**Introduction:**

Most patients are readily liberated from mechanical ventilation (MV) support, however, 10% - 15% of patients experience failure to wean (FTW). FTW patients account for approximately 40% of all MV days and have significantly worse clinical outcomes. MV induced inspiratory muscle weakness has been implicated as a contributor to FTW and recent work has documented inspiratory muscle weakness in humans supported with MV.

**Methods:**

We conducted a single center, single-blind, randomized controlled trial to test whether inspiratory muscle strength training (IMST) would improve weaning outcome in FTW patients. Of 129 patients evaluated for participation, 69 were enrolled and studied. 35 subjects were randomly assigned to the IMST condition and 34 to the SHAM treatment. IMST was performed with a threshold inspiratory device, set at the highest pressure tolerated and progressed daily. SHAM training provided a constant, low inspiratory pressure load. Subjects completed 4 sets of 6-10 training breaths, 5 days per week. Subjects also performed progressively longer breathing trials daily per protocol. The weaning criterion was 72 consecutive hours without MV support. Subjects were blinded to group assignment, and were treated until weaned or 28 days.

**Results:**

Groups were comparable on demographic and clinical variables at baseline. The IMST and SHAM groups respectively received 41.9 ± 25.5 vs. 47.3 ± 33.0 days of MV support prior to starting intervention, *P *= 0.36. The IMST and SHAM groups participated in 9.7 ± 4.0 and 11.0 ± 4.8 training sessions, respectively, *P *= 0.09. The SHAM group's pre to post-training maximal inspiratory pressure (MIP) change was not significant (-43.5 ± 17.8 vs. -45.1 ± 19.5 cm H_2_O, *P *= 0.39), while the IMST group's MIP increased (-44.4 ± 18.4 vs. -54.1 ± 17.8 cm H_2_O, *P *< 0.0001). There were no adverse events observed during IMST or SHAM treatments. Twenty-five of 35 IMST subjects weaned (71%, 95% confidence interval (CI) = 55% to 84%), while 16 of 34 (47%, 95% CI = 31% to 63%) SHAM subjects weaned, *P *= .039. The number of patients needed to be treated for effect was 4 (95% CI = 2 to 80).

**Conclusions:**

An IMST program can lead to increased MIP and improved weaning outcome in FTW patients compared to SHAM treatment.

**Trial Registration:**

ClinicalTrials.gov: NCT00419458

## Introduction

Failure to wean (FTW) from mechanical ventilation (MV) is a significant clinical and economic problem. In 2003, approximately 300,00 patients required MV support for more than 96 hours in the USA and the estimated cost of these episodes was $16 billion [1]. The number of patients requiring long-term MV support is increasing five times as rapidly as the number of hospital admissions [2] and many of these patients experience FTW.

The etiology of FTW is often complex, but an imbalance in the demand placed on the inspiratory muscles used to generate inspiratory pressure during tidal breathing and their maximal pressure generating capability (Pi_br_/Pi_max_) has been implicated as a major contributor to this problem [3-5]. Numerous animal studies have documented ventilator-induced diaphragm dysfunction following as little as six hours of controlled MV [6-8], but less data examining the effects of MV on the human diaphragm are available. Knisely et al. [9] studied two children who had been ventilated for 7 and 45 days and qualitatively found profound atrophy of diaphragm muscle fibers following prolonged MV support. Levine et al. [10] documented approximately 55% atrophy in human diaphragms following 19 to 56 hours of controlled MV. Hermans et al. [11] recently reported marked reductions in magnetically stimulated transdiaphragmatic pressure in humans in the first week of MV support. Hussain et al. documented upregulation of catabolic process in human diaphragms following 15 to 276 hours of controlled MV [12], and Jaber et al. documented a 32% reduction in endotracheal tube pressure following magnetic diaphragm stimulation in humans following six days of MV support [13].

As an elevated Pi_br_/Pi_max _ratio is thought to be a major contributor to weaning failure [4,5] and MV has been shown to rapidly cause diaphragm weakness in humans, strength training the inspiratory muscles emerges as a possible treatment for FTW. Preoperative inspiratory muscle strength training (IMST) has been shown to reduce the incidence of postoperative respiratory complications in high-risk cardiac surgery patients [14] and has also been demonstrated to preserve postoperative inspiratory muscle strength following major abdominal surgery [15].

We [16] and others [17,18] have published successful case series and Caruso et al. published an unsuccessful [19] trial examining the effect of IMST on weaning outcome in FTW patients, but to date no adequately powered, randomized trial examining the effect of IMST on weaning outcome exists. We hypothesized that an IMST program, grounded in accepted principles of muscle strength training [20], coupled with progressively lengthening breathing trials (BT) would improve weaning outcome compared with the SHAM condition.

## Materials and methods

After approval from the University of Florida Health Center Institutional Review Board (Federal wide Assurance FWA00005790), written informed consent was obtained from the patients or their legally designated surrogates. The trial was registered on Clinical Trials number NCT00419458. Patients were recruited from the adult medical, general surgical and burn ICUs of Shands Hospital at the University of Florida. Censuses of patients who were supported with MV were regularly queried and patients who had FTW with usual care were identified. Subjects were considered a FTW case when the patient failed to wean with usual care. Entry and exclusion criteria are shown in Table 1.

**Table 1:** Entry and exclusionary criteria

Age 18 years or older
Adequate gas exchange as indicated by a P_a_O_2 _above 60 mmHg while breathing with an F_I_O_2 _of 0.50 or less
Be medically stable and ready to be weaned from the ventilator as determined by the attending physician
Hemodynamically stable for 24 hours prior to participation or requiring only minimal intravenous pressor agents (dobutamine or dopamine ≤ 5 mcg/kg/min, phenyleprine ≤ 1 mcg/kg/min)
Be able to follow simple verbal directions related to inspiratory muscle strength testing and training
Receiving assist control or SIMV or pressure support ventilation via a tracheostomy, with SIMV ≤ 6 breaths/min, pressure support ventilation ≤ 15 cm H_2_O and PEEP ≤ 10 cmH_2_O
Unable to sustain unsupported breathing for at least 72 consecutive hours following resolution of factor(s) precipitating respiratory failure
Demonstrate normal hemidiaphragm positions on X-ray
Not have any progressive neuromuscular disease such as amyotrophic lateral sclerosis, muscular dystrophy, multiple sclerosis, myasthenia gravis, or any other neuromuscular disorder that would interfere with responding to inspiratory muscle training
Have an anticipated life expectancy of at least 12 months
Have a core temperature between ≥36.5°C and ≤ 38.5°C
Not have a spinal cord injury above T8
Not have any skeletal pathology (scoliosis, flail chest, spinal instrumentation) that would seriously impair the movement of the chest wall and ribs
Not using any type of home MV support prior to hospitalization
Body mass index < 40 kg/m^2^
Not require continuous sedative or analgesic agents that will depress respiratory drive or the ability to follow commands
No excessive secretions (requiring suctioning more than once every hour)
Not being considering for transfer to another hospital in the next
28 days

F_i_O_2_, fraction of inspired oxygen; MV, mechanical ventilation; P_a_O_2, _arterial pressure of oxygen; PEEP, positive end expiratory pressure; SIMV, synchronized intermittent mandatory ventilation.

Subjects were studied from February 2004 until February 2009. The protocol was a single-blinded design with SHAM treatment. Subjects were blinded to their group assignment. Randomization was performed with a computerized random number generator and group assignments were sealed in opaque envelopes. Subjects were not randomized until they failed the initial BT.

### Maximal inspiratory pressure measurement

Maximal inspiratory pressure (MIP) was measured on the first day of participation, every Monday and on days when the subjects attempted a 12-hour aerosol tracheotomy collar (ATC) trial. MIP was measured using the method of Caruso et al. [21]. Briefly, a one-way valve was attached to the patient's tracheostomy tube that allowed exhalation but blocked inspiration. The valve was connected to an electronic recording manometer and the patient was vigorously encouraged to inhale and exhale as forcefully as possible for 20 seconds. MIP measurements were repeated three times with a two-minute rest period with MV support between each attempt; the most negative value was recorded.

### Inspiratory muscle strength training

IMST was performed five days per week (Monday to Friday) with a threshold inspiratory muscle trainer (Threshold PEP; Respironics Inc; Murrysville, PA, USA), which provided a threshold inspiratory pressure load between -4 and -20 cmH_2_O. The Threshold PEP device is marketed as an expiratory positive pressure device, but can provide an inspiratory threshold load if one inspires through the exhalation port. An inspiratory threshold training device is commercially available (Threshold IMT; Respironics Inc; Murrysville, PA, USA), but we found that many patients were unable to open the poppet valve at the lowest pressure setting (8 cmH_2_O) on the Threshold IMT device. When performing IMST, the subjects were disconnected from the MV and the IMST device was attached to their tracheostomy tube with the cuff inflated. Subjects breathed room air during IMST. Subjects performed four sets of 6 to 10 breaths per day, with two minutes of rest with MV support between each set. The training device was set to the highest pressure setting that the subject could consistently open during inspiration, and was progressed daily as tolerated. Subjects were instructed to inhale and exhale as forcefully as possible during the IMST breaths. The IMST training program was based on clinical experience obtained prior to initiating this trial. Respiratory pressures at the tracheostomy tube were monitored during IMST and SHAM training with CO_2_SMO Plus respiratory monitors with Analysis Plus software (Respironics Inc; Murrysville, PA, USA) interfaced to a laptop computer.

### SHAM training

The SHAM group used a resistive inspiratory muscle training device (Pflex; Respironics Inc; Murrysville, PA, USA) set on the largest opening. The Pflex device had a 3 mm hole drilled into the body, which further reduced the pressure required to generate airflow. Subjects performed SHAM training by being removed from the ventilator circuit and the training device was attached to the tracheostomy tube. Subjects breathed room air during SHAM treatment. Subjects performed four sets of 6 to 10 breaths, five days per week, and were instructed to breathe with long, slow inspiratory and expiratory efforts during training. SHAM subjects were given two minutes of rest with MV support between each set. IMST and SHAM treatments were normally conducted between 07.30 am and 09.00 am, Monday through Friday.

### Breathing trials

All subjects participated in progressively lengthening BTs with reduced or no MV support. Three types of BT were used: ATC, continuous positive airway pressure (CPAP) and reduced pressure support trials. Trials were conducted seven days per week, usually commencing around 09.00 am and only one trial per day was attempted. The initial BT was an ATC trial, and patients were allowed to breathe without MV support as long as tolerated. Subjects who tolerated this initial ATC trial for 72 hours were considered weaned and were not studied. Criteria for terminating BT included: 30 beats/min or more increase in heart rate, systolic blood pressure above 180 mmHg or below 90 mmHg, oxygen-hemoglobin saturation (S_P_O_2_) below 90% for five minutes, respiratory rate above 35 breaths/min for five minutes, serious dysrhythmias, if the patient requested to be returned to MV support or there was clinical evidence of respiratory distress (substernal retraction and sternocleidomastoid retraction, paradoxical breathing, or diaphoresis).

The daily progression for the ATC trials was: one, two, three, four, six, nine, and twelve hours. The second ATC trial was targeted for the step below the duration the patient tolerated on their first ATC trial, not to exceed six hours. For example, if a patient tolerated four hours on the initial ATC trial, the second ATC trial duration was three hours, the next four hours and so on. When a subject failed an ATC trial, the next trial was the same duration. If a subject was unable to participate in ATC trials for several days, the ATC trial target duration was decreased by the number of steps equal to the number of days missed. When subjects successfully completed a 12-hour ATC trial, the next day they progressed to breathing without MV support as tolerated. If they tolerated the ATC trial for 72 hours, they were classified as weaned.

If the subject was unable to complete at least one hour on the initial ATC trial, the next day a one-hour CPAP trial was attempted. CPAP trials were progressed by one hour per day until reaching three hours and then the patient began the ATC trial schedule as above. If the patient was unable to complete the initial one-hour CPAP trial, the next day they attempted a one-hour reduced pressure support trial (no synchronized intermittent mandatory ventilation breaths, about 50% of their baseline pressure support and baseline positive end expiratory pressure (PEEP)). If successful, the reduced pressure support trial duration was increased by one hour per day until reaching three hours whereupon they then began the CPAP and ATC trial progressions as detailed above.

Patients received usual nursing care during BT, but rehabilitation activities were withheld during BT until the patients could tolerate a six-hour ATC BT. Once patients could tolerate a six-hour BT, rehabilitation activity during BT was begun but reduced to approximately 50% of the normal duration and intensity until weaning. Breathing data during BT were monitored with ICU clinical bedside monitors and with a CO_2_SMO Plus respiratory monitor with Analysis Plus software (Respironics Inc; Murrysville, PA, USA) interfaced to a laptop computer. Prior to commencing the first and final BT, dynamic compliance and inspired and expired airway resistance were measured with the CO_2_SMO Plus respiratory monitors while the patients received their baseline level of MV support.

### Statistical analysis

Categorical variables were analyzed with Chi-square tests. Between groups tests on continuous variables were analyzed with independent samples Student t tests. Within-group variables were analyzed with t tests for paired measures. Repeated measures analysis of variance (ANOVA) tests were used for variables with group, time factors, and group × times interactions. Cell means contrasts were used to explore differences when significant interactions were present in ANOVA. Statistical significance was set at *P*< 0.05.

## Results

**Figure 1:**
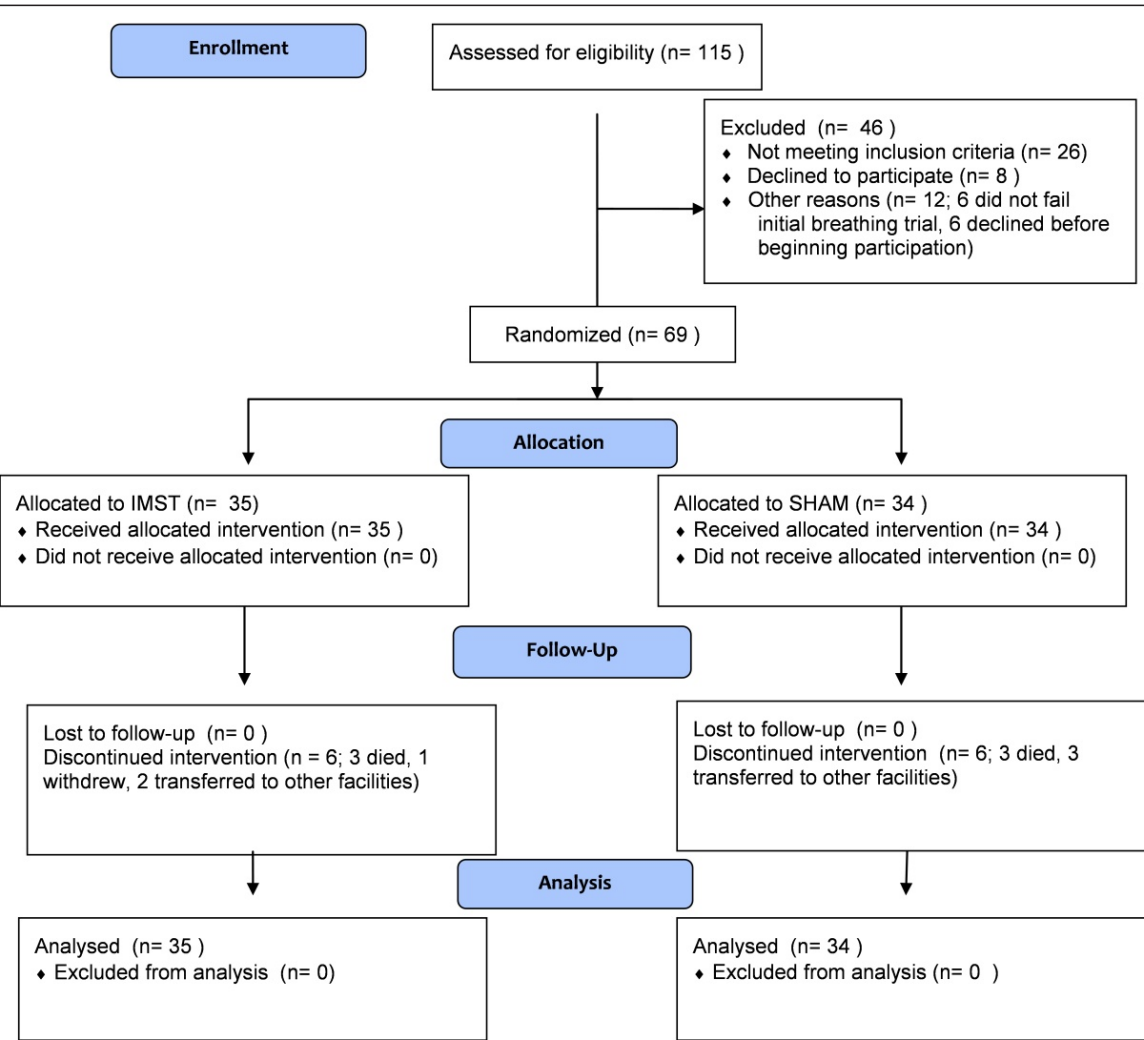
**CONSORT diagram**.

The flow of subjects from evaluation to participation is shown in the CONSORT diagram (Figure 1). The randomization process resulted in groups that were equivalent on demographic factors, reasons for respiratory failure, treatment with renal replacement therapy, duration of MV prior to starting study intervention, duration of the initial ATC trial to failure, MIP, and other prognostic variables (Tables 2 and 3). Additionally, both groups experienced similar comorbidities during hospitalization before intervention (Table 4), received similar pharmacologic management during study intervention (Table 5), experienced similar complications during the study (Table 6), and underwent similar diagnostic and therapeutic procedures during study intervention (Table 7). Of note, 43% of the IMST subjects and 29% of SHAM subjects were dialysis dependent. Dialysis dependency has been associated with a reduced wean rate [22,23].

**Table 2:** Primary admission medical and surgical diagnoses

Medical diagnosis	*IMST*	*SHAM*	*TOTAL*
***Cardiovascular***			
Acute congestive heart failure	1	.	**1**
Myocardial infarct or unstable angina	1	.	**1**
***Respiratory***			
Adult respiratory distress syndrome	3	.	**3**
Interstitial disease	1	.	**1**
Pneumothorax	.	1	**1**
Pulmonary vasculitis	.	1	**1**
***Neurological***			
Acute intracranial hemorrhage	1	.	**1**
***Gastrointestinal***			
Pancreatitis	1	1	**2**
***Infectious/metabolic***			
Sepsis with shock	2	2	**4**

***TOTAL MEDICAL PATIENTS***	10	5	**15**

**Surgical diagnosis**	*IMST*	*SHAM*	***TOTAL***

***Cardiovascular***			
Abdominal aortic aneurysm repair	2	2	**4**
Dissecting/ruptured aorta	1	1	**2**
Cardiac valve replacement	.	1	**1**
Peripheral artery bypass graft	1	.	**1**
Multiple simultaneous procedures	.	2	**2**
Other cardiovascular surgical procedures	2	.	**2**
***Gastrointestinal***			
Esophageal surgery - for neoplasm	5	2	**7**
Esophageal surgery - not for neoplasm	1	1	**2**
Gastrointestinal surgery - for neoplasm	.	1	**1**
Gastrointestinal surgery - not for neoplasm	6	6	**12**
Hepatobiliary surgery - for neoplasm	3	1	**4**
Hepatobiliary surgery - not for neoplasm	1	.	**1**
***Neurological***			
Craniotomy, not for neoplasm	.	4	**4**
Spinal surgery	.	2	**2**
Spinal cord injury	.	1	**1**
***Orthopedic***			
Orthopedic surgery, not hip replacement	.	2	**2**
Multiple simultaneous procedures	.	1	**1**
***Miscellaneous***			
Liver transplantation	2	1	**3**
Full-thickness burns/skin grafting	1	1	**2**

***TOTAL SURGICAL PATIENTS***	25	29	**54**

IMST, inspiratory muscle strength training.

**Table 3:** Demographic and medical data

	IMST *n *= 35	SHAM *n *= 34	*P *value
Age (years)	65.6 ± 11.7	65.1 ± 10.7	0.86
Gender (male/female)	16/19	15/19	0.42
Number of smokers Pack * years smoking history	12 54 ± 28	11 50 ± 30	0.86 0.72
Pre-albumin at study start (mg/dL)	15.3 ± 6.6	15.4 ± 6.3	0.96
MV support days to start of study intervention	41.9 ± 25.5	47.3 ± 33.0	0.36
Total MV support days from hospital admission until end of study participation	57.3 ± 29.5	63.5 ± 34.0	0.46
Total study days	14.4 ± 8.1	18.0 ± 8.8	0.10
SAPS II at study start	33.5 ± 8.6	33.0 ± 8.6	0.83
Dynamic compliance			
(ml/cm H_2_O)	*n *= 26	*n *= 27	^a^Tr = 0.93
Pre-training	53.9 ± 18.3	53.8 ± 17.1	^b^Ti = 0.19
Post-training	57.8 ± 19.5	57.1 ± 21.4	^c^Tr × Ti = 0.91
Dynamic inspired airway resistance			
(cm H_2_O/L/S)	*n *= 26	*n *= 27	^a^Tr = 0.70
Pre-training	7.8 ± 3.2	7.1 ± 3.0	^b^Ti = 0.12
Post-training	7.7 ± 1.8	8.8 ± 3.0	^c^Tr × Ti = 0.08
Dynamic expired airway resistance			
(cm H_2_O/L/S)	*n *= 26	*n *= 27	^a^Tr = 0.74
Pre-training	8.1 ± 3.6	7.3 ± 3.1	^b^Ti = 0.16
Post-training	7.9 ± 1.8	9.1 ± 3.4	^c^Tr × Ti = 0.07
***Renal function***			
Blood urea nitrogen (mg/dL)	35.6 ± 15.6	37.6 ± 23.3	0.67
Creatinine (mg/dL) (Includes subjects receiving renal replacement therapy)	1.1 ± 0.9	1.0 ± 0.7	0.74
Renal replacement therapy n (%)	15 (43%)	10 (29%)	0.33
Mean daily fluid balance (ml)	118 ± 964	405 ± 573	0.14
***Arterial blood gases on baseline MV support (initial day of study)***			
pH	7.41 ± 0.07	7.42 ± 0.06	0.68
P_a_CO_2 _(torr)	42.9 ± 7.4	40.3 ± 10.0	0.20
P_a_O_2 _(torr)	113.8 ± 48.0	108.8 ± 33.0	0.60
HCO_3_^- ^(mEq/L)	27.2 ± 5.1	26.0 ± 2.9	0.30
P_a_O_2_/F_i_O_2_	293 ± 125	278 ± 97	0.60
***MV settings (initial day of study)***			
SIMV (br/min)	4.5 ± 3.7	3.8 ± 2.2	0.37
Pressure Support (cm H_2_O)	10.4 ± 1.8	10.0 ± 3.4	0.53
PEEP (cm H_2_O)	5.4 ± 1.0	5.8 ± 1.6	0.27
F_i_O_2_	0.40 ± 0.03	0.40 ± 0.004	0.77
***Study-related activity***			
Initial ATC trial duration to failure (hours)	2.5 ± 2.1	3.1 ± 3.1	0.39
Number of study days patients were unable to participate	3.4 ± 5.0	3.8 ± 4.7	0.77
(% of study days)	(16 ± 21%)	(15 ± 18%)	0.87
Pressure setting on IMST device (cm H_2_O)	Pre 7.2 ± 2.6	-	< 0.0001
	Post 12.8 ± 3.6		
Pressure developed at tracheotomy tube during treatment (cmH_2_O)			^a^Tr = 0.26
Pre-training	-9.54 ± 3.70	-3.10 ± 1.54	^b^Ti = 0.0003
Post-training	-14.52 ± 4.59	-3.36 ± 2.08	^c^Tr×Ti < 0.0001

Data are mean ± standard deviation.

ATC, aerosol tracheotomy collar; F_i_O_2_, fraction of inspired oxygen; HCO_3_^-, ^arterial bicarbonate concentration; IMST, inspiratory muscle strength training; MV, mechanical ventilation; P_a_CO_2_, arterial pressure of carbon dioxide; P_a_O_2, _arterial pressure of oxygen; P_a_O_2_/F_i_O_2, _ratio of arterial pressure of oxygen to inspired oxygen fraction; PEEP, positive end expiratory pressure; SAPS II, new simplified acute physiology score; SIMV, synchronized intermittent mandatory ventilation.

^a^Tr, treatment factor, ^b^Ti, time factor, ^c^Tr × TI treatment × time interaction factor for two-way repeated measures analysis of variance on dynamic compliance, dynamic inspired airway resistance, dynamic expired airway resistance and pressure developed at tracheotomy tube during training variables. All other variables were tested with T tests for independent samples, paired samples or Chi-square tests.

**Table 4:** Comorbidities between hospital admission and entering study

	*IMST*	*SHAM*
***Cardiovascular***		
Angina	2	0
Atrial fibrillation	9	10
Bundle branch block	0	1
Arrhythmias requiring cardioversion	2	2
Congestive heart failure	7	5
Deep vein thrombosis	4	14
Cerebral vascular accident or intracranial hemorrhage	6	10
Myocardial infarction	6	5
Pacemaker	1	1
Pericarditis/endocarditis	0	1
Peripheral vascular disease/chronic wounds	3	7
***Respiratory***		
Adult respiratory distress syndrome	2	4
Aspiration pneumonia	9	6
Bronchitis/bronchiectasis/chronic obstructive pulmonary disease exacerbations	10	11
Pleural effusion	18	21
Pneumonia or tracheobronchitis	20	22
Pneumothorax	8	2
Pulmonary embolism	2	5
Hemothorax	3	2
Empyema	3	0
Respiratory arrest	1	2
Tracheal bleed	1	2
Bronchiolitis obliterans with organizing pneumonia	1	0
Cavitary respiratory lesions	1	1
***Metabolic/Endocrine***		
Adrenal depletion	1	1
Diabetes mellitus	11	9
Hypothyroidism	5	6
***Renal***		
Chronic renal failure (prior renal replacement therapy-dependence)	2	0
Acute renal failure (new renal replacement therapy dependence)	11	9
Renal insufficiency (no renal replacement therapy)	2	1
***Infections***		
Specific Organisms:		
*Candida albicans*	9	8
*Cytomegalovirus*	4	4
*Methicillin-resistant Staphylococcus aureus*	13	17
*Vancomycin-resistant enterococci*	8	6
*Pseudomonas*	13	13
Acid-fast bacillus smear positive	1	0
Indwelling line-associated sepsis	2	7
Urinary tract infection	13	10
Sepsis with shock	18	13
Sepsis without shock	5	6
***Gastrointestinal***		
Ascites	4	2
gastrointestinal hemorrhage	10	11
*Clostrium difficile colitis*	*5*	*4*
Ileus or gastroparesis	2	3
Necrotic bowel	4	1
Hepatic failure	0	1
Pancreatitis	5	1
Bowel perforation	1	1
Abdominal or peritoneal hematoma	5	3
Abdominal abscess	2	3
Necrotic gallbladder/cholelithiasis	3	2
Open abdomen	3	2
Abdominal compartment syndrome	0	1
***Organ transplantation***		
Liver	2	1
Cardiac	0	1
Renal	1	0
***Other***		
Cardiac arrest	6	7
Shock	0	2
New cancer diagnosis	11	9
Encephalitis	0	1
Encephalopathy (unspecified etiology)	5	1
Status epilepticus	0	1
Subacute or chronic fractures	1	3
Amputation	0	1
Wound	8	10
Wound or incisional dehiscence	4	5
Myoclonus	0	1
Critical illness myopathy (per physician)	3	1
Critical illness myopathy (per diagnostic test)	2	0

IMST, inspiratory muscle strength training.

**Table 5:** Drug use during intervention by group

	*IMST*	*SHAM*	*P value*
*Anabolic steroids*			
n (%)	6 (17%)	9 (26%)	*0.34*
Mean drug days	10.8 ± 4.8	15.6 ± 7.3	*0.19*
*Antibacterial agents*			
n (%)	30 (86%)	29 (85%)	*0.77*
Mean drug days	28. ± 27.8	31.0 ± 23.5	*0.71*
*Antiviral agents*			
n (%)	3 (9%)	1 (3%)	-
Mean drug days	16.3 ± 4.0	6	-
*Anti-arrhythmia agents*			
n (%)	13 (37%)	9 (26%)	*0.34*
Mean drug days	16.1 ± 11.0	14.8 ± 10.7	*0.77*
*Anti-hypertensive agents*			
n (%)	17 (49%)	20 (59%)	*0.47*
Mean drug days	13.8 ± 11.7	15.3 ± 12.9	*0.74*
*Bronchodilators*			
n (%)	16 (46%)	20 (59%)	*0.28*
Mean drug days	12.2 ± 7.5	17.3 ± 10.6	*0.43*
*Corticosteroids*			
n (%)	16 (46%)	13 (38%)	*0.53*
Mean drug days	12.2 ± 7.5	10.8 ± 14.4	*0.72*
*Diuretics*			
n (%)	21 (60%)	23 (68%)	*0.47*
Mean drug days	10.6 ± 6.6	11.0 ± 9.4	*0.88*
*Anti-glycemic agents*			
n (%)	24 (69%)	28 (82%)	*0.18*
Mean drug days	13.5 ± 9.1	14.3 ± 11.1	*0.77*
*Immune suppression agents*			
n (%)	3 (9%)	3 (9%)	*0.70*
Mean drug days	10.3 ± 14.4	3.7 ± 3.8	*0.48*
*Neuromuscular blockers*			
n (%)	1 (3%)	1 (3%)	-
Mean drug days	2.0	2.0	-
*Narcotic analgesic agents*			
n (%)	30 (86%)	26 (76%)	*0.33*
Mean drug days	12.4 ± 9.8	13.9 ± 8.7	*0.55*
*Sedatives*			
n (%)	27 (77%)	24 (71%)	*0.42*
Mean drug days	13.4 ± 13.0	11.3 ± 9.1	*0.50*
*Vasopressors*			
n (%)	5 (14%)	8 (24%)	*0.33*
Mean drug days	3.8 ± 4.7	3.1 ± 3.8	*0.78*
*Beta-blockers*			
n (%)	31 (89%)	29 (85%)	*0.96*
Mean drug days	19.6 ± 20.9	22.0 ± 21.6	*0.66*

IMST, inspiratory muscle strength training; n, number of subjects taking that category of drug, followed by the percent of the group taking that drug category. *P *values for proportions were calculated with chi square, corrected with Yate's correction for cells with five or less subjects. Mean drug days = mean number (± standard deviation) of drug days for the subjects taking that category of drugs. For example, if a patient took two different antibiotics for four days, that patient would have accumulated eight drug days for the antibiotic category. Drug days were tested with unpaired T tests.

**Table 6:** Complications occurring during intervention period

	*IMST*	*SHAM*
***Cardiovascular***		
New angina diagnosis	1	1
Deep vein thrombosis	1	4
Myocardial infarction	0	2
Pericardial effusion	0	2
Hypertensive crisis	10	4
***Respiratory***		
Aspiration pneumonia	3	1
Pneumonia or tracheobronchitis	13	13
Pneumothorax	0	1
Pleural effusion	9	3
Mucus plug	1	3
Other	2	7
***Infections***		
*Vancomycin-resistant enterococci*	11	4
*Methicillin-resistant staphylococcus aureus*	6	4
*Cytomegalovirus*	1	2
Indwelling line-associated sepsis	6	5
Urinary tract infection	13	7
Sepsis with shock	6	2
Sepsis without shock	6	7
Other	10	9
***Gastrointestinal***		
Ascites	1	0
*Clostridium difficile colitis*	2	0
Gastrointestinal hemorrhage	7	6
Hepatic failure	0	1
***Renal***		
Acute renal insufficiency	1	0
Renal failure	2	1
***Other***		
Tracheal bleeding	2	4
Cardiac arrest/cardiopulmonary resuscitation	2	4
Death	3	3

IMST, inspiratory muscle strength training.

**Table 7:** Diagnostic and therapeutic procedures performed during study

	*IMST*	*SHAM*
***Imaging***		
Computerized tomography scan	23	18
Echocardiogram	4	3
Endoscopy, lower	0	2
Endoscopy, upper	3	3
Diaphragm movement test	2	2
Electroencephalogram	0	1
Venous Doppler test	10	9
Other	7	6
***Lines and Tubes***		
Abdominal drain	10	1
Central venous catheter	22	19
Chest tube	23	26
Gastrostomy tube	7	10
Jejunostomy/gastro jejunum tube	14	8
Peripherally inserted central catheter line	35	47
Other	28	33
***Medical therapy***		
Bronchoscopy	26	42
Renal replacement treatments	111	62
Thoracentesis	3	0
Transfusion, blood (units)	81	132
Transfusion, other blood products	6	35
Wound debridement	1	1
Other	8	14
***Surgery***		
Abdominal	3	1
Head/neck	1	3
Vascular	1	1
Thoracic	0	3
Other	3	5

IMST, inspiratory muscle strength training.

Six subjects did not fail during the initial ATC trial and were weaned without further intervention. These subjects were not randomized to treatment groups and were not included in the analysis. Three IMST subjects died during the 28-day treatment period, one withdrew from the study and two patients were transferred to other facilities before completing 28 days of treatment. These six subjects were classified as weaning failures. Three subjects in the SHAM group died during the 28-day treatment period and three subjects were transferred to other facilities before completing 28 days. These six subjects were also classified as weaning failures.

Excluding the initial BT, the IMST group performed 330 trials and the SHAM group performed 382 trials. The IMST and SHAM groups successfully completed 77.0% and 73.0% of the BT, respectively (*P *= 0.23).

The IMST and SHAM groups participated in 9.7 ± 4.0 and 11.0 ± 4.8 strength and SHAM training sessions, respectively (*P *= 0.09). The mean training pressure setting on the IMST device was 7.2 ± 2.6 vs. 12.8 ± 3.6 cmH_2_O for the initial and final training bouts, respectively (*P*< 0.0001, Table 3). The SHAM group's modified training device was set at the largest orifice (lowest resistance setting) for all sessions. The IMST group developed -9.54 ± 3.70 and -14.52 ± 4.59 cmH_2_O of inspiratory pressure at the tracheotomy tube during the initial and final IMST bouts (*P *= 0.0004). Corresponding training pressure values for the SHAM group were -3.10 ± 1.54 and -3.36 ± 2.08 cmH_2_O (*P *= 0.86). The treatment × group interaction for pressure developed during training was significant (*P*< 0.0001). The SHAM group's pre to post-training MIP change was not significant (-43.5 ± 17.8 vs. -45.1 ± 19.5 cmH_2_O, *P *= 0.39), while the IMST group's MIP increased (-44.4 ± 18.4 vs. -54.1 ± 17.8, cmH_2_O, *P*< 0.0001). There were no adverse events observed during IMST or SHAM treatments.

Twenty-five of 35 IMST subjects weaned (71%, 95% confidence interval (CI) = 55% to 84%), while 16 of 34 (47%, 95% CI = 31% to 63%) SHAM subjects weaned (*P *= 0.039). The number of patients needed to be treated for effect was 4 (95% CI = 2 to 80).

In order to further explore the role of MIP changes in weaning outcome, we performed a *post-hoc *analysis on MIP using weaning outcome as the independent measure. The pre- and post-training MIP measures for the weaning success (*n *= 41) and failure (*n *= 28) groups were respectively; -44.0 ± 20.2 and -53.5 ± 20.7 cmH_2_O versus -43.9 ± 14.8 and -43.9 ± 15.0 cmH_2_O. A repeated measures ANOVA revealed a significant outcome × time interaction and the change in MIP for the successfully weaned group was significantly greater than the failure to wean group (*P*< 0.0001).

## Discussion

Our primary findings were that the IMST rehabilitation program rapidly improved MIP and improved weaning outcome compared with the SHAM condition. The weaning rate (47%) achieved by the SHAM group was comparable with usual care conditions as reported in observational studies examining comparable FTW patients [24-26].

Other workers have shown that MIP is a poor predictor of extubation success [27-31]. Several differences between this study and the studies that found MIP to be a poor predictor of extubation outcome must be acknowledged: 1) studies that have shown MIP to be a poor predictor of extubation outcome examined intubated patients in the acute phase of MV support [28,31], whereas our subjects had received MV support for approximately six weeks prior to starting intervention and all of our patients had tracheotomies; 2) our selection criteria identified patients who had FTW because of inspiratory muscle weakness that was amenable to strength training; and 3) none of the studies that have evaluated MIP as an extubation predictor used any type of strength training program to increase MIP. Investigators have found that higher values of MIP are associated with improved weaning outcome in chronic FTW patients. Yang [31] reported in a cross-sectional study that the Pi_br_/Pi_max _ratio of successfully weaned patients was lower than FTW patients. Carlucci et al. [5] have recently shown in an observational study with a group of long-term FTW patients similar to ours, that patients who eventually weaned, improved their MIP, and lowered the Pi_br_/Pi_max _ratio, while those who FTW did not. Our findings also support a role for increased MIP in improved weaning outcomes.

We propose that respiratory muscle weakness is a greater contributor to failed weaning than fatigue. During failed BTs in FTW patients, respiratory distress is often clinically described as "fatigue". However, several authors have reported heightened respiratory muscle activity during failed BTs compared with stable respiratory muscle activity among patients who successfully completed BTs. For example, Teixeira et al. [32] measured a 50% increase in the work of breathing of FTW patients over the course of failed BTs, whereas successful patients maintained a constant work of breathing during the trials. Jubran et al. [33] reported similar findings and an absence of low-frequency fatigue during failed BT.

Alternatively, we hypothesize that inspiratory muscle weakness initiates a high proportional ventilatory drive requirement during unassisted BT, when weakened inspiratory muscles must generate increased muscle tension in order to adequately ventilate the lungs. During MV support, a relatively low motor drive elicits large, ventilator-assisted tidal volume breaths. When an unsupported BT begins, a discrepancy between the elevated respiratory drive and afferent lung volume feedback can lead to an increased awareness of respiratory effort [34]. Perceived feedback errors will be addressed by further increases in respiratory motor drive, but the feedback discrepancy cannot be corrected by a highly-driven, weakened inspiratory pump that generates insufficient volume feedback [35].

Ongoing efferent-afferent feedback errors propel a positive feedback loop, resulting in the progressively higher levels of respiratory drive, inspiratory esophageal pressure, and work of breathing reported by others, and it may lead to clinical respiratory distress [34,36]. If this positive feedback cycle progresses to high levels of inspiratory muscle work, reflex sympathetic activation can occur, with shunting of blood from the periphery to the working respiratory muscles [37,38]. Elevated sympathetic activity is a probable cause of the tachycardia, hypertension, and diaphoresis frequently observed during failed BT in FTW patients. IMST has been shown to attenuate the sympathetic activation induced by high intensity inspiratory muscle work [39].

Strengthening the inspiratory muscles theoretically could correct the feedback discrepancy between respiratory drive and lung/chest expansion and may result in a lower perception of breathing effort. The perception of breathing effort has been experimentally altered by manipulations of inspiratory muscle strength. Campbell et al. [40] studied the perception of inspiring against standard inspiratory resistive loads before and after weakening the inspiratory muscles to about 30% of baseline with neuromuscular blockade. In the weakened state, subjects rated the effort of loaded breathing higher than in the unblocked condition. We [41] studied the effects of strengthening the inspiratory muscles on perception of inspiratory effort and respiratory drive in healthy subjects. Both the respiratory drive and the effort of breathing against standard inspiratory resistive loads were lower following a 50% improvement in MIP. These findings support the hypothesis that the perception of inspiratory effort and respiratory drive are inversely proportional to inspiratory muscle strength and may help explain why an increased MIP contributed to weaning.

Whenever severely debilitated patients undergo muscle strength training, the possibility of exercise-induced muscle damage must be considered. Human [42,43] studies have documented that long-term, high resistance inspiratory loading can induce diaphragm muscle fiber damage. Although we did not examine diaphragm samples for training-induced damage, we think that it is unlikely that the IMST program induced muscle damage for the following reasons: 1) the duration of muscle loading during each IMST training session was approximately one minute per day. In contrast, animal and human studies have documented diaphragm damage with prolonged, high resistance loads, lasting 1.5 [44,45] to 96 hours [46]. 2) Our IMST patients were able to inspire against increasing inspiratory loads on a daily basis. If the patients had been experiencing muscle soreness and contractile fiber damage from IMST, one would have expected diminished muscle performance, rather than increasing performance.

Our results are encouraging, but limitations must be acknowledged. The weaning results were significant, but this was a single site study with a relatively small sample size. Our IMST method is not suitable for all FTW patients. Patients must be sufficiently alert to cooperate with IMST, and patients whose FTW etiology is not the result of treatable inspiratory muscle weakness are unlikely to benefit from IMST. Our subjects were recruited primarily from surgical ICUs, with approximately 22% of the subjects treated in the medical ICU.

## Conclusions

In conclusion, we found an improved MIP and weaning outcome with IMST compared with SHAM training in medically complex, long-term FTW patients. IMST is a clinically practical and safe method to improve weaning outcome in selected FTW patients.

## Key messages

• IMST can rapidly increase MIP in medically complex, long-term FTW patients.

• IMST, in conjunction with BT, can increase the number of FTW patients weaned versus SHAM training plus BT.

## Abbreviations

ANOVA: analysis of variance; ATC: aerosol tracheotomy collar; BT: breathing trials; CI: confidence interval; CPAP: continuous positive airway pressure; FTW: failure to wean; IMST: inspiratory muscle strength training; MIP: maximal inspiratory pressure; MV: mechanical ventilation; PEEP: positive end expiratory pressure; Pi_br_/Pi_max_: ratio of inspiratory tidal breathing pressure to maximal inspiratory pressure; S_p_O_2_: oxygen-hemoglobin saturation.

## Competing interests

The University of Florida and Drs Martin, Gabrielli and Banner have applied for a patent to modify clinical mechanical ventilators to provide threshold inspiratory muscle training to patients receiving mechanical ventilation support.

## Authors' contributions

ADM had full access to all of the data in the study and takes responsibility for the integrity of the data and the accuracy of the data analysis. ADM, Gabrielli, MBanner, LJC, PD, EH and RJG contributed to study concept and design. ADM, BKS, TH and HD contributed to acquisition of data. ADM, AG, PD, MBanner, EH, MBaz, RJG and BKS contributed to analysis and interpretation of data. ADM, BKS, MBanner, RJG and AJL contributed to drafting of the manuscript. AG, PD, MBanner, EH, MB, HD, TH, RJG and AJL contributed to critical revision of the manuscript for important intellectual content. ADM, BKS, HD and TH contributed to statistical analysis. ADM obtained funding. This project was supported by NIH R01HD42705 to ADM. AJL, MBanner, LJC and MBaz contributed to administrative, technical, or material support. ADM, AG, LJC, EH, AJL, MBaz and RJG contributed to study supervision.
